# Thermophysical Properties of Larch Bark Composite Panels

**DOI:** 10.3390/polym13142287

**Published:** 2021-07-12

**Authors:** Lubos Kristak, Ivan Ruziak, Eugenia Mariana Tudor, Marius Cătălin Barbu, Günther Kain, Roman Reh

**Affiliations:** 1 Faculty of Wood Sciences and Technology, Technical University in Zvolen, 96001 Zvolen, Slovakia; kristak@tuzvo.sk (L.K.); ruziak@tuzvo.sk (I.R.); reh@tuzvo.sk (R.R.); 2 Forest Products Technology and Timber Construction Department, Salzburg University of Applied Sciences Markt 136a, 5431 Kuchl, Austria; cmbarbu@unitbv.ro (M.C.B.); gkain.lba@fh-salzburg.ac.at (G.K.); 3 Faculty of Furniture Design and Wood Engineering, Transilvania University of Brasov, B-dul. Eroilor nr. 29, 500036 Brasov, Romania

**Keywords:** larch bark, thermophysical properties, insulation materials, ecofriendly composites, tannin-based adhesive

## Abstract

The effects of using 100% larch bark (*Larix decidua* Mill) as a raw material for composite boards on the thermophysical properties of this innovative material were investigated in this study. Panels made of larch bark with 4–11 mm and 10–30 mm particle size, with ground bark oriented parallel and perpendicular to the panel’s plane at densities varying from 350 to 700 kg/m^3^ and bonded with urea-formaldehyde adhesive were analyzed for thermal conductivity, thermal resistivity and specific heat capacity. It was determined that there was a highly significant influence of bulk density on the thermal conductivity of all the panels. With an increase in the particle size, both parallel and perpendicular to the panel´s plane direction, the thermal conductivity also increased. The decrease of thermal diffusivity was a consequence of the increasing particle size, mostly in the parallel orientation of the bark particles due to the different pore structures. The specific heat capacity is not statistically significantly dependent on the density, particle size, glue amount and particle orientation.

## 1. Introduction

Buildings are one of the most contributing sectors to energy consumption (residential/commercial). In the European Union (EU), the building sector is responsible for over 40% of overall energy consumption, which makes it a significant contributor to CO_2_ emissions [1,2,3,4]. Due to the rising energy consumption of buildings (residential/commercial), increased prices of fossil fuels and its effect on global warming, energy saving through the use of thermal insulation is regarded as an effective and efficient method [5,6]. Efficient thermal insulation is a highly relevant topic [7,8,9], as highlighted by the fact that in the EU the heating of living space corresponds to the main use of energy in houses (64% of total energy consumption in houses) [10,11].

The current trends in residential construction can be simply characterized as the trends of increasing quality requirements for structures connected to a growing awareness towards using materials with less economic and environmental impact [12,13,14]. With environmental concerns related to buildings, sustainability is a relevant topic in the construction industry [15], which results in growing pressure to evaluate the environmental impacts of currently used and alternative materials to achieve lower impact. Sustainability is also an important part of new EU strategy [16,17], which solves the problems in the area of recycled economy. Solid waste recovery belongs to main strategies for a circular economy [18].

Current insulation materials such as polystyrene, polyisocyanurate, and PUR foams in the construction market are generally based on fossil resources with a great insulation performance but with high environmental impact due to their production processes [19,20,21,22].

The use of natural or recycled materials for these purposes is an emerging trend in this area to warrant a healthy environment. Some of these materials are already on the market while others are still at an early stage of production or development study [23,24]. Many natural products were investigated and tested for use as insulation materials such as kenaf, flax, bamboo fibers, corn husk, reeds, straw, rice husk, hemp, wood fibers, etc. [25,26,27,28,29,30,31,32,33,34,35,36]. These materials have great potential because of low density, low environmental impact and appropriate thermal properties [37,38]

During harvesting of wood, a significant proportion of bark accrues. The harvested bark amounts up to 10% of biomass on average. It means worldwide between 0.16 and 0.19 billion cubic meters of bark annually come from the wood industry [39,40]. Tree bark, as natural barrier material, can be used as default material for insulation purposes [41]. Bark is suitable for thermal insulation material due to its low density [42], very good thermal insulation properties, high proportion of cork cells [43], high chemical extractives content serving as a protection against microorganisms [44,45] and low flammability [46,47]. Moreover, the bark-based composites are included in the category of products with very low formaldehyde emissions [48,49].

The aim of this study was to investigate the thermophysical properties of larch bark composites with densities ranging from 350 to 700 kg/m^3^, by determining the thermal conductivity, thermal diffusivity and specific heat capacity.

## 2. Materials and Methods

### 2.1. Material

The raw material for the manufacturing of the panels was larch bark (*Larix decidua* Mill.), from Graggaber larch sawmill in Unternberg, Salzburg County, with a specific gravity of 0.32 [42]. The bark planks were dried by means of a vacuum kiln dryer type Brunner–Hildebrand High VAC-S, HV-S1 (Hannover, Germany) from 90% to 9% moisture content, at a temperature of 60 °C and 200–250 mbar pressure. Two categories of particle sizes were obtained by crushing of massive bark in a 4-spindle shredder type RS40 from Untha (Kuchl, Austria). The particle grain sizes are: 4–11 mm and 10–30 mm. Urea-formaldehyde (UF) adhesive Prefere 10F102 from Metadynea (Krems, Austria) (66% solid content, pH 8.3–9 and viscosity 60–90 mPa*s) was used for the binding of larch bark particles. The resination factor is presented in Table 1, together with the experimental design.

The bark particles were blended with UF in a plough share mixer ENT type WHB-75. The mixture was introduced in a 320 × 320 × 30 mm^3^ mold and consequently pressed in a hydraulic laboratory press type Höfer HLOP 280 press (Taiskirchen, Austria). The plate temperature was 180 °C and the press factor 20 s/mm (in this case significantly higher than in an industrial application). Before testing, the samples were conditioned for one month at 20 ± 1 °C temperature and 65 ± 1% air humidity to obtain standard climatizing for all studied samples. The testing specimens for determining thermal conductivity and diffusivity were cut according to EN 326-1. (European Committee for Standardization: EN 326-1:2005 Wood based panels—Sampling, cutting and inspection—Part 1: Sampling and cutting of test pieces and expression of test results. 2005, Brussels, Belgium).

The thermal measurements were performed on 8 different bark-based composites (Table 1). The size of samples was 100 × 100 × 20 mm^3^. The design of the experiment included four independent parameters, i.e., density *ρ* (under Archimedes law), resination factor RF, particle size *PS* and particle orientation PO. The density was calculated after climatization.

### 2.2. Methods

**Figure 1:**
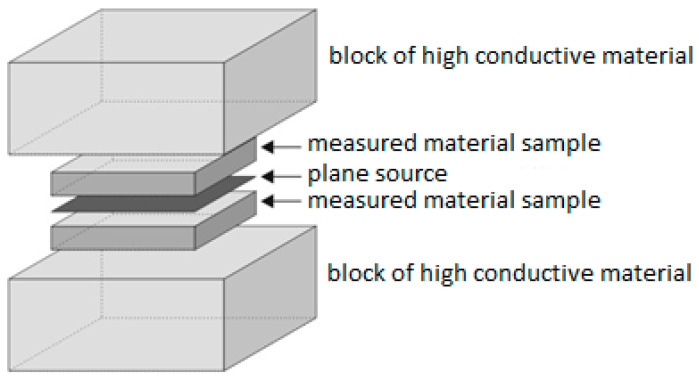
The EDPS apparatus scheme.

The thermal conductivity, thermal diffusivity and specific heat capacity were determined using the EDPS method [50,51]. The extended dynamic plane source (EDPS) method involves two samples of low conductive material (lambda value lower than 2 W/m*K) localized between two large blocks of high conductive material. Between them is localized a heat plane source and the measuring sensor (Figure 1).

The plane source (Figure 1) is measuring changes of electrical resistance R and computed temperature from the linear relation between electrical resistance and temperature, which for the plane source is characterized by the resistance temperature coefficient 4.8 × 10^−3^ Ω.K^−1^ and the resistance at 20 °C which is equal to 3.6 Ω. The EDPS method is suitable for low conductive samples and the experimental set-up is made in such a manner that the heat flux is one directional through sample thickness, which in all measuring cases was fulfilled.

The EDPS method computed the thermal conductivity from temperature difference and thermal diffusivity *a* from the fitted value of characteristic time τ which characterizes the temperature increase velocity and is defined by the Formula (1). The measuring accuracy of the method is also verified by the variance coefficient.

(1) $$\tau =\frac{l^{2}}{a}$$

where:

l—sample thickness in m,
a—thermal diffusivity of material in m^2^.s^−1^.

The theoretical temperature time dependence is defined by Formula (2)

(2) $$T\left(t\right)=\frac{q\times l}{\lambda }\times \sqrt{\frac{t}{\pi \times \tau }}\times \left(1+2\sqrt{\pi }\sum_{n=1}^{\infty }\beta^{n}\times ierfc\left(n\times \sqrt{\frac{\tau }{t}}\right)\right)$$

where:

*q*—heat flux density in W.m^−2^
*l*—sample thickness in m
*λ*—thermal conductivity of sample in W.m^−1^.K^−1^
*t*—time in s
τ—relaxation time in s
ierfc—error function

Every sample was measured 5 times. For each type of board 10 measurements were performed. The measuring characteristics were determined at 0.6 A for 300 s. After measuring thermal conductivity and thermal diffusivity, specific heat capacity was computed by Equation (3):

(3) $$c=\frac{\lambda }{a\times \rho }$$

where:

λ—material thermal conductivity in W.m^−1^.K^−1^,
a—thermal diffusivity of material in m^2^.s^−1^,
ρ—material density in kg.m^−3^.

The thermophysical properties of bark-based materials can be measured by EDPS method with very high repeatability. The validity of the EDPS method was characterized by the coefficient of correlation with theoretical function, which for all measurements was higher than 0.9995.

## 3. Results and Discussion

### 3.1. Thermal Conductivity

The thermal conductivity of all panels was significantly affected by panel density, particle size and particle orientation. These variables have a statistically significant influence. The regression analysis was made for both particle orientations, parallel and perpendicular to panel´s plane. There is a strong correlation of the panel´s thermal conductivity with density in all cases (R = 0.99). In addition, regression coefficients are significant for all regression functions. For analysis, the dataset was divided into 2 independent categories, one for perpendicular board orientation (PP) and another for parallel board orientation (PA). The dataset for horizontal direction is shown in Table 2 and for vertical direction in the Table 3.

The multiple regression was performed to analyze the effect of the chosen input parameters on thermal conductivity (*TC*) with Statistica software (version 12) to determine the regression constants and also the sensitivity coefficients of the input parameters in relation to the curve fitting, which finds the only regression constants with 95% CI boundaries. From the measured and analyzed results it can be concluded that the resination factor did not have significant effect on *TC*.

Based on the values of input parameters and *TC* as the output parameter, the regression dependence of thermal conductivity *TC_PA_* (parallel to the panel´s plane) as a polynomial function of particle size *PS* and density *ρ* was determined in the form (4):

(4) $$TC_{PA}=7.90\times 10^{-5}\times \rho +1.65\times 10^{-4}\times PS+0.0357$$

In the same manner, the regression dependence of thermal conductivity *TC_PE_* (perpendicular to the panel´s plane) was calculated as a function of particle size *PS* and density *ρ* for perpendicular board direction in the form (5)

(5) $$TC_{PE}=11.0\times 10^{-5}\times \rho +3.65\times 10^{-4}\times PS+0.0390$$

By means of multiple regression, the sensitivity coefficients of particle size *PS* and density *ρ* on thermal conductivity in both parallel and perpendicular directions were calculated also, which are shown in Table 4.

This research showed a highly significant (*p* < 0.001) influence of bulk density on the thermal conductivity of boards in all cases. The low density of the insulation boards is directly correlated with the lowering thermal conductivity due to a higher porosity and high void content in the boards [52,53]. The thermal conductivity of the porous material is a combination of conduction (solid part) and convection (gaseous part) [54]. The air present in the internal voids of an insulating material offers high thermal resistance due to its low conductivity. In the case of the small pores in panels, the air in the voids is static and the convection effect is minor. The results concerning the density and porosity influence on the thermal conductivity were confirmed for bark insulation board by [55,56], and also for fiberboards [57,58], OSB [51] and other insulation boards [59]. An interesting phenomenon was observed for insulating materials by Haupl [60], where the decrease of the thermal conductivity diminishes with decreasing density for very low densities. This phenomenon was observed also in the case of wood-based loose-fill thermal insulation materials [61], textiles [62] or hemp fibers [63].

A significant increase (*p* < 0.001) of thermal conductivity with increasing particle size in both perpendicular and parallel orientation was found. The reason is due to the different pore structures. The heat flow is transferred through the solid substance and voids filled with air, while the thermal conductivity of the air inside the voids is much lower than in the solid material (voids serve as scattering centers for phonons and they take up a fraction of the heat conduction volume of the material). In the case of fine fraction of the bark boards, the pores are smaller and closed. That is why the static and convection effect is minor compared to the coarse fraction boards at the same density [6,52,64,65].

The findings of this study showed the strong influence of the orientation of the particles. The average increase of *TC* in the perpendicular direction versus the parallel direction of the particles to the panel´s plane is almost 20%, which implies that the bark particle orientation is an important factor when producing insulation panels with specific characteristics. The main reason can be explained using the theory of optimal heat conduction pathways for external porosity materials, which is bonded above by the effective medium theory (EMT) equation, and below by the Maxwell–Eucken equation with the lower-conductivity material as the continuous phase [66]. The particles form conducts the heat flow, resulting in higher thermal conductivity. Another explanation is that the particles oriented perpendicularly to the panel´s plane have the higher compaction of particles, which results in higher density areas within the panel structure [67]. Kain et al. [68] examined larch bark insulation boards and showed a significant influence of the particle orientation on the thermal conductivity. The thermal conductivity of bark-based panels with particles oriented parallel to the plane was on average 16% lower than of boards with perpendicular particles. In the above-mentioned study, particles were used with sizes between 10 and 30 mm and a moisture content of 8%. The authors found the dependencies for parallel and perpendicular directions in the forms (6) and (7):

(6) $$TC\left(PA\right)=9.5.10^{-5}\times \rho +0.03525$$

(7) $$C\left(PE\right)=12.9.10^{-5}\times \rho +0.03575$$

From comparison of Equations (4) and (6) for the parallel direction and Equations (5) and (7) for the perpendicular direction it is clear that slopes of Equations (3) and (4) are significantly higher, which is logical because there is no function of particle size. However, the ratio of density slopes for parallel direction vs. perpendicular direction is equal to 0.737 for Equations (6) and (7) and 0.718 for the model with particle sizes *PS*. The difference between these two ratios is equal to 2.55%, which is the result of the different particle sizes in Equations (4) and (5). The average absolute part for the density-particle size model for both directions is equal to 0.0373 W.m^−1^.K^−1^ and for the density only model equal to 0.0355 W.m^−1^.K^−1^, which means that the deviation then is equal to 5.15%. Similar results for the influence of the orientation of particles to *TC* were confirmed in the case of bark boards also in [68]. This theory was also confirmed for wood fiberboards [57,69] and other wood based composites [70].

The density–particle size model was verified also in the study of [55]. According to this reference, the slope of thermal conductivity versus density for all directions is equal to 8.4 × 10^−5^ W.m^2^.K^−1^.kg^−1^. For comparison, the slope of the experimental results is equal to 7.97 × 10^−5^ W.m^2^.K^−1^.kg^−1^ which corresponds to 5.1%, which means very good agreement.

Another important parameter is the thickness of the boards. In the case of the samples with vertical orientation, with the thickness reduction the thermal conductivity values decrease very slowly. For boards with thickness bellow 20 mm, the thermal conductivity might be significantly influenced by the coarse particles cavities, which reach nearly from one side to another resulting in worse conductivity values due to the convection of air, which is able to transfer a significant amount of thermal energy inside the panel. This phenomenon will diminish with increased thickness of the boards [71,72,73].

### 3.2. Thermal Diffusivity

The relation between thermal diffusivity, thermal conductivity, specific heat and density is well known. In the case of density and specific heat these properties may be considered isotropic. On the other hand, thermal conductivity and thermal diffusivity are anisotropic.

From measured and analyzed results it can be concluded that the resination factor does not have significant effect on thermal diffusivity. Based on the values of input parameters and thermal diffusivity as output parameters, the regression dependence of thermal diffusivity *α_PA_* was determined as a function of particle size *PS* and density *ρ* in the form (8)

(8) $$a_{PA}=0.218-1.98\times 10^{-4}\times \rho -4.39\times 10^{-4}\times PS$$

The regression dependence of thermal diffusivity *α_PE_* as a function of particle size *PS* and density *ρ* for perpendicular board direction is shown in the form (9):

(9) $$a_{PE}=0.254-2.15\times 10^{-4}\times \rho -5.00\times 10^{-4}\times PS$$

By means of multiple regression, we also calculated the sensitivity coefficients of particle size *PS* and density *ρ* on thermal diffusivity in both directions, which are shown in Table 5.

The decreasing trend of thermal diffusivity *a* vs. density is in good agreement with work by [74], which predicted the non-linear decrease of the thermal diffusivity vs. density of bark. The result is also in agreement with [75], in the case of wood-based sandwich panels for use as structural insulated walls and floors. As shown in research of [76], thermal diffusivity of wood in air decreases with density, but on the other hand under vacuum conditions the values of thermal diffusivity (vs. density) are almost the same. This explains our theory, that low density and therefore high porosity (high void content) of a material is a predominant factor of thermal diffusivity dependence. This theory also explains a significant decrease (*p* < 0.001) of thermal diffusivity with increasing particle size in both perpendicular and parallel orientation (again in as in the case of thermal conductivity) due to the different pore structures.

From Table 5 it can be concluded that the thermal diffusivity of samples with particles oriented perpendicular to the panel´s plane is more strongly affected by the particle size. This finding is correct because thermal diffusivity corresponds to thermal wave propagation velocity through the object. In the particles perpendicular to the panel´s plane orientation, the thermal conductivity and thermal diffusivity are higher than in the parallel direction. For larger particle sizes, the process of temperature equaling in the volume is slower because the larger particles need more time to equal its temperature to the temperature of surroundings created by air capsules, thus the thermal diffusivity is lower.

The authors in [55] listed the intervals of thermal diffusivity of bark. The minimal value of bark thermal diffusivity is equal to 0.107 mm^2^.s^−1^ and maximal value of bark thermal diffusivity is equal to 0.214 mm^2^.s^−1^. All measured and predicted values (based on Equations (8) and (9)) of thermal diffusivities in this study are within this interval. The lowest values of predicted thermal diffusivity can be found for density 700 kg.m^−3^ and coarse particles (0.108 for parallel direction and 0.116 mm^2^.s^−1^ for perpendicular direction). On the other hand, the highest values of predicted thermal diffusivity can be found for density 300 kg.m^−3^ and fine particles (0.192 for parallel direction and 0.208 mm^2^.s^−1^ for perpendicular direction).

Comparison of the predicted thermal diffusivities in both directions leads to 10.6% higher absolute value of thermal diffusivity in the perpendicular direction. The slopes for the perpendicular direction for density and *PS* are 8.6% and 14% higher versus the parallel direction.

### 3.3. Specific Heat Capacity

Specific heat capacity is defined as the amount of energy needed to increase 1 kg of mass one unit in temperature (K). The heat capacity of wood depends on the temperature and moisture content of the wood but is practically independent of density or species [77,78]. The specific heat of coniferous tree bark (oven-dry at 22 °C) in the literature varies from 820 J.kg^−1^.K^−1^ to almost 1400 J.kg^−1^.K^−1^ [79,80]. In some research there was a significant variation between trees, but not the variation between species [74], in most of research there is no significant difference between specimens or species [81].

According to experimental results the specific heat capacity in perpendicular direction is equal to $c_{PE}=\left(1391.5\pm 24.2\right)\;J.{\mathrm{kg}}^{-1}.K^{-1}$ and for parallel direction equal to $c_{PA}=\left(1377.8\pm 32.3\right)\;J.{\mathrm{kg}}^{-1}.K^{-1}$. The percentual deviation between the directions is equal to 0.99%, which is well under the specific heat capacity uncertainty, therefore we can conclude that the measured results are in very good agreement with theory. In the next step, we verified the dependence of specific heat capacity versus density. The coefficient of determination R^2^ was 0.03, therefore it can be concluded that the density has no significant effect on the value of specific heat capacity, which again is in agreement with theory. Similar results were achieved for particle size (R^2^ = 0.15).

After concluding that the specific heat capacity is not statistically significant dependent on the density, particle size, glue amount and particle orientation it was finally computed the average value of this parameter which is equal to $c=\left(1382.9\pm 28.5\right)\;J.{\mathrm{kg}}^{-1}.K^{-1},$ which is in good agreement with the literature values.

### 3.4. Bark Panels vs. Other Insulation Panels

This research showed that density, orientation of the particles and particles size have strong influence on the thermal conductivity and thermal diffusivity of investigated panels. The best results were measured for boards with densities of about 350–400 kg/m^3^ with thermal conductivity in the range 0.065–0.070 W.m^−1^.K^−1^, thermal diffusivity in the range of 0.13–0.17 mm^2^.s^−1^ and specific heat capacity of around 1300 J.kg^−1^.K^−1^.

The thermal conductivity of bark boards is comparable to other insulation panels (Figure 1). These panels can be divided into conventional (such as expanded polystyrene, extruded polystyrene, stone wool, glass fibers). These materials offer many different options with easy installation and have excellent thermal properties. On the other hand, these materials have lower mechanical properties as bark boards and are derived from petrochemical substances and are an environment threat [82].

Another group of thermal insulation panels consists of natural materials (natural or recycled, such as hemp, flax, sheep wool, jute fiber, wood fiber, rice straw, cotton). These materials are very popular because they are renewable, cheap and have low health risk during processing. Their tendency to absorb a large amount of moisture [83] is problematic, as is their low fire and anti-fungal/bacteria resistance [84,85,86]. These problems can be solved by chemical treatment of the surface, which lowers the environmental advantage of these materials [87,88,89,90].

Bark has, besides low density, excellent thermal properties and very good acoustic properties together with natural resistivity against microorganisms and fire [53,91,92,93]. Another great advantage of bark is its property as a formaldehyde scavenger [94,95,96]. From an economic point of view, bark as a by-product of timber manufacturing and is available at low prices.

**Figure 2:**
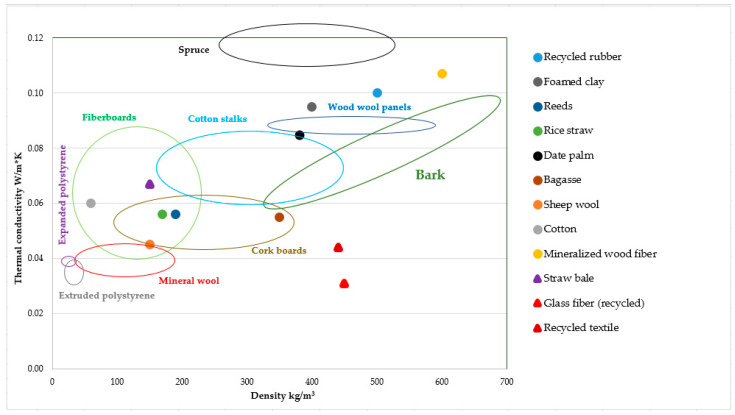
Thermal conductivity of thermal insulation boards.

**Figure 3:**
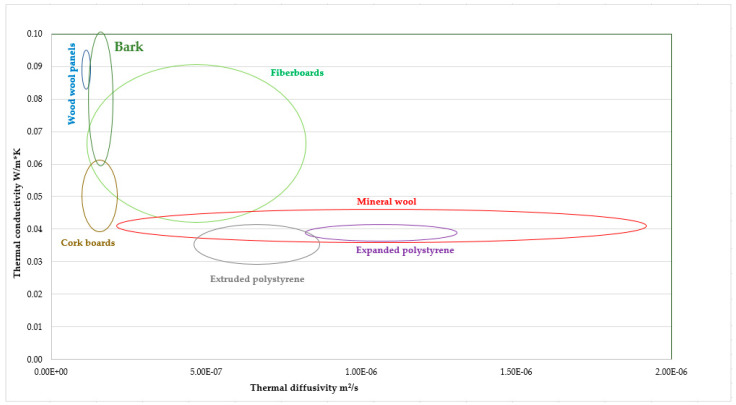
Thermal conductivity vs. thermal diffusivity of insulation materials.

Bark insulation boards have comparable thermal conductivity to most natural insulation materials (Figure 2). Excellent thermal insulation also requires very good thermal diffusivity properties. In this area, bark insulation boards belong to the best materials due to very low thermal diffusivity (Figure 3). Bark boards panels with these parameters provide very good thermal inertia and heat storage capacity, all together with good thermal insulation compared to other insulation materials [97]. This combination reduces the extreme values of temperature inside a building as compared to materials with higher densities which provide less thermal insulation [98,99].

## 4. Conclusions

The findings of this study have shown a highly significant influence of bulk density on the thermal conductivity of all boards. The increasing in particle size both parallel and perpendicular to the panel´s plane directions determined accordingly an increase in thermal conductivity. The average increase of the thermal conductivity in the perpendicular direction versus the parallel direction of the particles to the panel´s plane is almost 20%, which implies that the bark particle orientation is an important factor when producing insulation panels with specific characteristics.

A significant decrease was determined of thermal diffusivity with the increase particle size, mostly in the parallel orientation of the bark particles due to the different pore structures. For samples with particles oriented perpendicular to the panel´s plane, the particle size was observed as a stronger influence on the thermal diffusivity. Perpendicular to the panel´s plane orientation of particles direction, the thermal conductivity and thermal diffusivity are higher than in the parallel direction.

The specific heat capacity is not statistically significantly dependent on the density, particle size, glue amount and particle orientation.

The present study showed that the larch bark composites could be successfully used for thermal insulation in constructions.

## Figures and Tables

**Table 1:** Design parameters of measured materials.

**Material**	**1**	**2**	**3**	**4**
Image	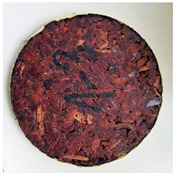	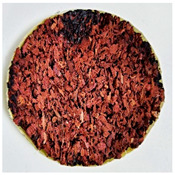	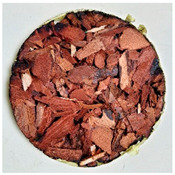	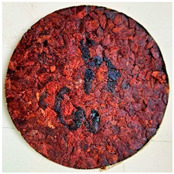
Density [kg.m^−3^]	688±14	345±7	47±25	537±11
Resination factor [%]	10	10	10	10
Particle size [mm]	4–11	4–11	10–30	4–11
Particle orientation	parallel	perpendicular	parallel	parallel
**Material**	**5**	**6**	**7**	**8**
Image	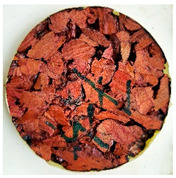	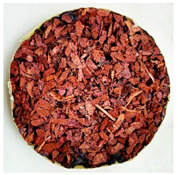	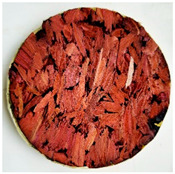	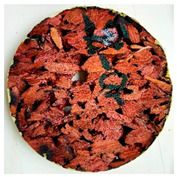
Density [kg.m^−3^]	369±12	362±9	471±6	355±8
Resination factor [%]	20	10	10	20
Particle size [mm]	10–30	4–11	10–30	10–30
Particle orientation	parallel	parallel	perpendicular	perpendicular

**Table 2:** Measured average values of input and output parameters for the boards with particles oriented parallel to the panel´s plane.

Sample	Glue Amount (%)	Density (kg.m^−3^)	Particle Size (mm)	Thermal Conductivity (W.m^−1^.K^−1^)	Specific Heat Capacity (J.kg^−1^.K^−1^)	Thermal Diffusivity (mm^2^.s^−1^)
1	10	688	4 to 11	0.107	1400	0.111
3	10	477	10 to 30	0.067	1392	0.101
4	10	537	4 to 11	0.071	1321	0.100
5	20	369	10 to 30	0.071	1380	0.139
6	10	362	4 to 11	0.065	1395	0.129

**Table 3:** Measured average values of input and output parameters for the boards with particles oriented perpendicular to the panel´s plane.

Sample	Glue Amount (%)	Density (kg.m^−3^)	Particle Size (mm)	Thermal Conductivity (W.m^−1^.K^−1^)	Specific Heat Capacity (J.kg^−1^.K^−1^)	Thermal Diffusivity (mm^2^.s^−1^)
2	10	345	4 to 11	0.078	1373	0.165
7	10	471	10 to 30	0.104	1382	0.160
8	20	355	10 to 30	0.081	1418	0.161

**Table 4:** Sensitivity coefficients for *TC* for both vertical and horizontal directions.

Sensitivity of *TC* on	Parallel Direction	Perpendicular Direction
Density (kg.m^−3^)	90.4%	74.6%
Particle size (mm)	9.6%	25.4%

**Table 5:** Sensitivity coefficients for thermal diffusivity for both directions.

Sensitivity of *a* on	Parallel Direction	Perpendicular Direction
Density (kg.m^−3^)	89.9%	80.7%
Particle size (mm)	10.1%	19.3%

## Data Availability

Not applicable.
